# Risk Factors and Predictive Modeling for Post-Acute Sequelae of SARS-CoV-2 Infection: Findings from EHR Cohorts of the RECOVER Initiative

**DOI:** 10.21203/rs.3.rs-2592194/v1

**Published:** 2023-03-08

**Authors:** Chengxi Zang, Yu Hou, Edward Schenck, Zhenxing Xu, Yongkang Zhang, Jie Xu, Jiang Bian, Dmitry Morozyuk, Dhruv Khullar, Anna Nordvig, Elizabeth Shenkman, Russel Rothman, Jason Block, Kristin Lyman, Yiye Zhang, Jay Varma, Mark Weiner, Thomas Carton, Fei Wang, Rainu Kaushal, RECOVER Consortium

**Affiliations:** Weill Cornell Medicine; Weill Cornell Medicine; Cornell University; Cornell University; Cornell University; Cornell University; Cornell University; Cornell University; Cornell University; Cornell University; Cornell University; Cornell University; Cornell University; Cornell University; Cornell University; Weill Cornell Medicine; Weill Cornell Medicine; Weill Cornell Medicine; Weill Cornell Medicine

## Abstract

**Background:**

Patients who were SARS-CoV-2 infected could suffer from newly incidental conditions in their post-acute infection period. These conditions, denoted as the post-acute sequelae of SARS-CoV-2 infection (PASC), are highly heterogeneous and involve a diverse set of organ systems. Limited studies have investigated the predictability of these conditions and their associated risk factors.

**Method:**

In this retrospective cohort study, we investigated two large-scale PCORnet clinical research networks, INSIGHT and OneFlorida+, including 11 million patients in the New York City area and 16.8 million patients from Florida, to develop machine learning prediction models for those who are at risk for newly incident PASC and to identify factors associated with newly incident PASC conditions. Adult patients aged 20 with SARS-CoV-2 infection and without recorded infection between March 1^st^, 2020, and November 30^th^, 2021, were used for identifying associated factors with incident PASC after removing background associations. The predictive models were developed on infected adults.

**Results:**

We find several incident PASC, e.g., malnutrition, COPD, dementia, and acute kidney failure, were associated with severe acute SARS-CoV-2 infection, defined by hospitalization and ICU stay. Older age and extremes of weight were also associated with these incident conditions. These conditions were better predicted (C-index >0.8). Moderately predictable conditions included diabetes and thromboembolic disease (C-index 0.7–0.8). These were associated with a wider variety of baseline conditions. Less predictable conditions included fatigue, anxiety, sleep disorders, and depression (C-index around 0.6).

**Conclusions:**

This observational study suggests that a set of likely risk factors for different PASC conditions were identifiable from EHRs, predictability of different PASC conditions was heterogeneous, and using machine learning-based predictive models might help in identifying patients who were at risk of developing incident PASC.

## Plain Language Summary

Can we predict those who are at risk of newly incident post-acute sequelae of SARS-CoV-2 infection (PASC) using EHR data and machine learning predictive models? What are the factors associated with newly incident PASC conditions?In this retrospective cohort study of 35,275 (22,341) adults with SARS-CoV-2 infection and 326,126 (177,010) adults without SARS-CoV-2 infection from INSIGHT (OneFlorida+), we found that several incident PASC conditions (e.g., malnutrition, COPD, dementia, and acute kidney failure) were associated with severe acute SARS-CoV-2 infection and older age, and these conditions were better predicted based on information collected before baseline and during the acute phase. However, a variety of PASC conditions were moderately predictable (e.g., diabetes and thromboembolic disease) or less predictable (e.g, fatigue, anxiety, sleep disorders, and depression).These findings suggest that different PASC conditions exhibited heterogeneous predictability and association patterns and using machine learning-based predictive models and EHR datasets could help in identifying patients who were at risk of developing incident PASC conditions and managing the heterogeneous PASC conditions.

## Introduction

The global COVID-19 pandemic starting in late 2019 has led to more than 557 million infections and 6.4 million deaths as of July 14, 2022.^1^ Growing scientific and clinical evidence has demonstrated the existence of potential post-acute and long-term effects of COVID-19, which affect multiple organ systems^2^ and are referred to as post-acute sequelae of SARS-CoV-2 infection (PASC). Recently there have been several retrospective cohort analyses identifying potential PASC using real-world patient data^3(p19),4,5(p19)^. However, research on the predictability of PASC and their associated risk factors is still limited, and mixed results have been reported. Such predictive modeling research can help patients and healthcare professionals to recognize the risk of PASC early and inform effective actions. Several studies found older age, higher severities in the acute phase of SARS-CoV-2 infection^6^, and pre-existing conditions (e.g., hypertension, obesity) may be associated with a higher risk of developing PASC.^7–12^ By contrast, some studies also reported that baseline clinical characteristics or demographics were not associated with PASC.^10^ Two main challenges may explain these seemingly conflicting findings: 1) Prior studies have typically been conducted using patient cohorts with small sample sizes including only a few hundred^13^ or thousand^8^ patients, limiting the significance and generalizability of conclusions derived; and 2) PASC conditions are highly heterogeneous^14–16^, thus their predictabilities and associated risk factors could be heterogeneous as well.

To fill in the knowledge gap and address these challenges, we conducted a systematic study on the predictability of a broad spectrum of incident PASC conditions and their associated risk factors. We used two large electronic health records (EHR) cohorts from the PCORnet clinical research networks (CRN)^17^, namely INSIGHT^18^, covering patients in the New York City (NYC) area, and OneFlorida +^19^, including patients from Florida. The INSIGHT and OneFlorida + were used as primary analyses and validation respectively. A wide range of PASC conditions were selected based on our previous findings using a rigorous data-driven analysis pipeline^14^ and other existing evidence or clinical knowledge (See the method section for a detailed list of PASC diagnoses). We developed machine learning-based prediction models to identify patients who were more likely to develop particular incident PASC conditions with their baseline characteristics and acute severity according to medical utilization. We compared the performance of machine learning models with different levels of complexity, including regularized Cox proportional hazard model, regularized logistic regression, gradient boosting machine, and deep neural network in both the survival analysis setting and binary classification setting, as well as examined the potential risk factors for different PASC conditions after removing background associations. We observed that within the broad range of PASC, a pattern of post-severe acute disease-associated conditions was reliably predictable. Decreasing the burden of severe disease will likely improve these outcomes. However, a variety of PASC conditions were less predictable and were less associated with upfront disease severity. This lack of predictability may represent a challenge as the burden of severe disease decreases. This study is part of the NIH Researching COVID to Enhance Recovery (RECOVER) Initiative, which seeks to understand, treat, and prevent the post-acute sequelae of SARS-CoV-2 infection (PASC).

## Methods

### Data

This study used two large-scale de-identified real-world EHR datasets from the INSIGHT Clinical Research Network (CRN)^18^ and the OneFlorida + CRN^19^. The INSIGHT CRN contained longitudinal clinical data of approximately 12 million patients in the New York City metropolitan area, and the OneFlorida + CRN contained the EHR data of nearly 15 million patients from Florida and selected cities in Georgia and Alabama. The use of the INSIGHT data was approved by the Institutional Review Board (IRB) of Weill Cornell Medicine following NIH protocol 21–10-95–380 with protocol title: Adult PCORnet-PASC Response to the Proposed Revised Milestones for the PASC EHR/ORWD Teams (RECOVER). The use of the OneFlorida + data for this study was approved under the University of Florida IRB number IRB202001831.

### Definition of Post-acute Sequelae of SARS-CoV-2 (PASC)

We examined a list of potential PASC conditions as outcomes, including depressive disorders, anxiety disorder, general PASC symptoms and signs with ICD codes U099/B948, fever, malaise and fatigue, dizziness, malnutrition, fluid disorders, diabetes mellitus, edema, pressure ulcers, hair loss, paresthesia, dermatitis, chronic obstructive pulmonary disease (COPD), atelectasis, pulmonary fibrosis, dyspnea, acute pharyngitis, acute bronchitis, dementia, myopathies, cerebral ischemia, encephalopathy, cognitive problems, sleep disorders, headache, muscle weakness, fibromyalgia, joint pain, acute kidney failure, cystitis, genitourinary problems, constipation, gastroparesis, abdominal pain, gastroesophageal reflux disease (GERD), heart failure, hypotension, pulmonary embolism, thromboembolism, abnormal heartbeat, chest pain, and anemia. We compiled this list based on both our previous study and evidence from other literature.^3,4,14^ Any incident condition was defined in the SARS-CoV-2 infected patients who had the condition from 31 days to 180 days after but not having the condition three years to seven days before their acute infection. See Supplementary Table 1 for the detailed code list.

### Eligibility criteria and study population

**Figure 1:**
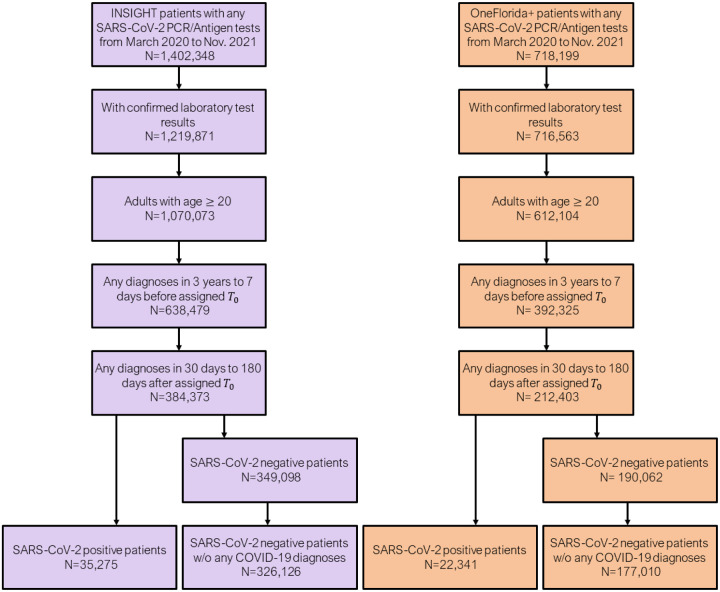
Patient inclusion and exclusion cascades of both the INSIGHT and OneFlorida+ cohorts, March 2020 to November 2021.

We included adult patients aged 20 years or older with at least one SARS-CoV-2 polymerase chain reaction (PCR) or antigen laboratory test from March 1st, 2020, to November 30th, 2021. We further required at least one diagnosis code within three years to seven days before the index date (referred to as the baseline period), and at least one diagnosis code from 31 days to 180 days after the index date (referred to as the post-acute phase or follow-up period), to ensure that patients were connected to the healthcare system and were being observed during the study period. We followed each patient from 31 days after his/her index date until the day of the first target outcome, documented death, the latest date of any documented records in the database, 180 days after the baseline, or the end of our observational window (December 31, 2021), whichever came first. Two exposure groups were the SARS-CoV-2 infected group and the non-infected group. The SARS-CoV-2 infected group included patients with a positive SARS-CoV-2 PCR or antigen laboratory test. The index date of the infected group was defined as the date of the first documented positive PCR or antigen test. The non-infected group included patients whose SARS-CoV-2 PCR or Antigen tests were all negative throughout the entire study period with no documented COVID-19-related diagnoses at any time. The index date for patients in the non-infected group was defined as the date of the first negative PCR or antigen test. The association study and predictive modeling were conducted on the infected group. The non-infected group was used to rule out background associations that were not specific to PASC. The patient inclusion and exclusion cascades were illustrated in Fig. 1.

### Covariates

We collected clinical features in the baseline period (3 years to 1 week before lab-confirmed SARS-CoV-2 infection) and the severity of acute infection (1 week before to 30 days after lab-confirmed SARS-CoV-2 infection). Age was categorized into 20–39 years, 40–54 years, 55–64 years, 65–74 years, and 75 years and older groups. We set 55–64 as the reference group. Gender was grouped into female and male (reference). Only three patients in INSIGHT were identified as other/missing gender who were excluded. The race was categorized into Asian, Black or African American, White (reference), other or missing. Ethnicity was grouped into Hispanic, not Hispanic (reference), and other/missing. We used the national-level area deprivation index (ADI) to capture the socioeconomic disadvantage of patients’ residential neighborhood.^20^ Larger ADI values indicate mode socioeconomically deprived status. Missing ADI value was imputed with median ADI per site. The ADI is a ranking from 1 to 100 with 1 and 100 representing the lowest and the highest level of disadvantage, respectively. We grouped ADI into five categories and set the ADI category 1–20 as the reference group. Baseline healthcare utilization up to three years before the index date was measured according to their care setting. For each inpatient, outpatient, and emergency department encounter, we categorized each setting into 0 visit (reference group), 1 or 2 visits, and 3 or more visits, respectively. We also considered the infection periods, which were grouped into March 2020 – June 2020, July 2020 – October 2020, November 2020 - February 2021, March 2021 – June 2021, and July 2021 – November 2021. We set the first wave of the pandemic from March 2020 to June 2020 in INSIGHT as the reference group. Of note, the third wave from July 2021 – November 2021 period was dominated by the Delta variant. Body mass index (BMI) was grouped according to the WHO classification, BMI < 18.5 as underweight, BMI 18.5–24.9 as normal weight (reference), BMI 25–29.9 as overweight, BMI > = 30 obese, and set missing value as a separate group. Smoking status was also considered, and categorized into never (reference), current, former, and missing. There is a significant missingness regarding smoking status (90.2% in COVID-positive patients and 49.8% in patients with at least one PASC) and we grouped these patients into the missing category.

A wide range of baseline clinical comorbidities were collected, based on a revised list of the Elixhauser comorbidities, conditions recommended by our clinician group, and related medications. Patients were ascertained as having a condition if they had at least two corresponding diagnoses documented during the baseline period. We also counted the number of pre-existing conditions and grouped them into no comorbidity (reference), 1, 2, 3, 4, and 5 or more. A detailed list of these pre-existing conditions and defined reference categories were summarized in Extended Data Table 1.

### Statistical analysis

For each potential PASC condition, we performed statistical analysis on its association with various covariates including the following steps.

- (Step I) We built a separate multivariate Cox proportional hazard model on the EHR of patients who were infected by SARS-CoV-2 to assess associations of covariates and time to the first incident event or censoring in the follow-up period (31–180 days after COVID-19 confirmation). The censoring event is defined as the earliest event of documented death, loss of follow-up in the database, 180 days after the baseline, or the end of our observational window (December 31, 2021). Fully adjusted hazard ratios (aHR) of each covariate and target PASC event were estimated.
- (Step II) We built another multivariate Cox proportional hazard model on the EHR of all patients regardless of their SARS-CoV-2 infection status. The model inputs include two parts. One is the set of covariates. The other is the set of interaction terms defined as the product of each covariate and SARS-CoV-2 infection status (1 for SAR-CoV-2 infected patients and 0 for non-infected control patients) on the outcome condition. In this way, the coefficient of a particular covariate captured its association with the outcome condition for patients who were not infected by SARS-CoV-2, and the coefficient of its corresponding interaction term captured the “quantitative modifications” of such association for patients who were infected by SARS-CoV-2. Fully adjusted hazard ratios of each covariate and interaction term were estimated on both infected and non-infected patients.

We identified a covariate to be a potential risk factor of a particular PASC condition if it satisfied the following three criteria:

- The adjusted hazard ratio (aHR) estimated from the infected patients in Step I were greater than 1;
- The p-value of the above aHR was smaller than 0.000562, which was corrected by the Bonferroni method for multiple testing;
- The aHR of the interaction term of the corresponding covariate should also be greater than 1 in Step II.

To build predictive models for each PASC condition, we examined different machine learning models in both survival analysis and binary classification settings. For the survival analysis setting, we used a multivariate Cox proportional hazard model with L2 norm regularization to predict the time to the outcome event. For the binary classification setting, the occurrence of the target event in the follow-up period was labeled as 1 and 0 otherwise. We used logistic regression with L2 norm regularization, gradient boosting machine with random forest base learner, and deep feed-forward neural network. For each of the abovementioned models, the best model was selected by grid search (see details in the following sensitivity analysis paragraph) a predefined hyper-parameter space through repeated cross-validation (ten times, five folds). The concordance index (C-index) and the area under the receiver operating characteristic curve (AUROC) were used to evaluate survival prediction performance and binary prediction performance respectively. Both two measures range from 0 to 1 with 0.5 indicating random guess and 1 indicating perfect prediction. The 95% confidence interval of the final performance was estimated by 1000-times bootstrapping performance on each of the testing datasets in repeated cross-validation.

### Stratified analysis

The stratified analysis was conducted by stratifying patients by their severity in the acute infection phase (hospitalized or non-hospitalized) and then performing statistical analysis within each stratum. The noninfected control patients were also stratified according to the hospitalized or non-hospitalized during the 1 week before to 30 days after their index date, to capture background associations within each subgroup population.

### Sensitivity analysis

To get robust conclusions, we conducted the following sensitivity analyses. For association analysis, we also used a univariate Cox model for each covariate adjusted for age, sex, and acute severity. We further tested the impact of lifting Step II of the statistical analysis on the identified risk associations.

For the predictive modeling, we also tested a different feature engineering method, which used the first 3-digits of ICD-10 codes and medication at the ingredient level to test to what extent PASC can be predicted in a data-driven manner. We selected 1,593 ICD-10 diagnosis codes, 2,309 drugs and 1,698 ICD-10 diagnosis codes, and 4,366 drugs from the INSIGHT dataset and OneFlorida + data, respectively. These ICD-10 diagnosis codes and medications were selected to construct the input feature vectors of the prediction model based on the significant difference (P-value less than 0.0001) between patients with positive and negative PASC conditions results. After the feature selection process, the selected ICD-10 diagnosis codes, medication, and collected baseline covariates were constructed to represent every PASC condition.

We also tested different machine learning predictive models in both the survival analysis setting and binary classification setting to validate the predictability of each PASC potentially impacted by different models. For the survival analysis setting, we tested Cox proportional model with L2-norm regularization. For the binary classification setting, we investigated three machine-learning models with different complexity. The first one is the regularized logistic regression. We adopted the L2-norm penalty and searched the inverse of regularization strength from 10^−3 to 10^3 with 0.5 as the sampling step size. The second one is the gradient boosting machine with a random forest as the base learner. We searched hyperparameters from maximum depth (3,4,5), max number of leaves in one tree (10,20,30), and a minimal number of data in one leaf (30). The third one is the deep forward neural network. We used ReLU (Rectified Linear Unit) activation function for the hidden layer, and search the hidden layers ((32,), (64,), (128,), (32, 32), (64, 64), (128, 128)), and learning rate (0.001, 0.01, 0.1). For each of the above-mentioned models, the best model was selected by grid search of the corresponding hyperparameter space through repeated cross-validation (ten times, five folds). In the repeated cross-validation process, we set one of the folds as the test set and the rest of the data as the training set. The C-index and the area under the receiver operating characteristic curve (AUROC) were used to measure the predictive performance in the survival setting and binary classification setting, respectively.

### Validation analysis and generalizability

To get a generalizable conclusion, we further replicated the abovementioned association analyses and predictive analyses to the OneFlorida + cohort. The cohort selection and modeling strategies were the same as our primary analyses on the INSIGHT cohort.

## Results

### Prediction Performance

We developed our primary results on the INSIGHT cohort and used the OneFlorida + cohort as a validation cohort. Both cohorts were collected from patients who has at least one PCR/antigen test for SARS-CoV-2 infection from March 2020 to November 2021, and the inclusion-exclusion cascade was provided in Fig. 1. The INSIGHT cohort included 35,275 adult patients with lab-confirmed SARS-CoV-2 infection and 326,126 non-infected control patients.

The current definition of PASC in the RECOVER protocols is ongoing, relapsing, new symptoms, or other health effects occurring four or more weeks after the acute phase of SARS-CoV-2 infection.^21^ We compiled a broad list of potential PASC conditions in terms of Clinical Classifications Software Refined (CCSR) categories^22^ based on our previous findings^14^ and evidence from other literature.^3,4^ Here we studied incident PASC conditions ascertained from 31 days to 180 days after the start of the acute SARS-CoV-2 infection date, denoted as the index date, but not existed one week to three years prior to the index date (See Method for the approach on how the list was compiled and Supplementary Table 1 for the detailed information).

**Figure 2:**
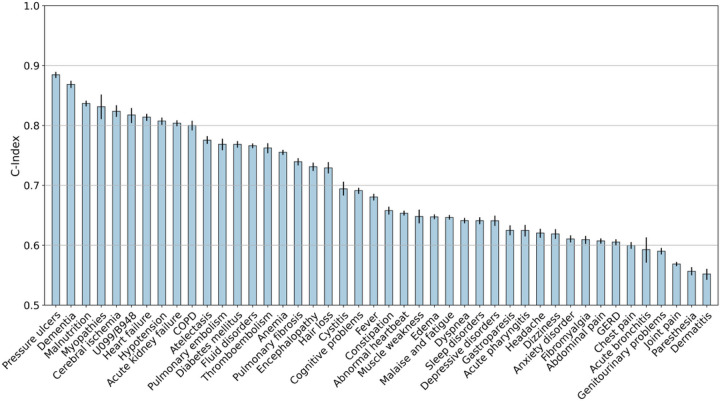
Prediction performance of different incident potential PASC conditions from baseline characteristics and the severity in the acute phase with the INSIGHT cohort from March 2020 to November 2021. The C-index with a 95% confidence interval was reported.

We built a list of 89 covariates that are potentially associated with PASC based on a revised list of Elixhauser comorbidities, recommendations of our RECOVER clinician team, and the severity of acute infection of SARS-CoV-2. These covariates included basic demographics (e.g., age, gender, race, ethnicity), social-economic status in terms of Area Deprivation Index (ADI)^21^, healthcare utilization history, body mass index, the period of infection, comorbidities, and the care settings in acute phase including hospitalization and ICU admission. For each of the categorical covariates, we defined its reference group the same as prior studies for acute SARS-CoV-2 infection (details see Method covariates section).^6^ We built different machine learning models to predict the individual risk of encountering each incident condition using these covariates. The prediction performance of a regularized Cox model measured by the Concordance index (C-index)^23^ with a 95% confidence interval was shown in Fig. 2 (results for other machine learning models are provided in the Sensitivity Analysis section).

Figure 2 shows that different incident conditions were associated with heterogeneous predictive performance. Conditions such as dementia, malnutrition, stroke, non-specific PASC (U099/B948), and kidney failure had a C-index > 0.8, in addition to other conditions such as myopathy, and pressure sores. We noted that diabetes, thromboembolic disease, and COPD were moderately predictable, with a C-index > 0.7, and other conditions such as fatigue, anxiety disorders, and sleep disorders were less predictable, with a C-index < 0.6.

#### Associations between risk factors and specific PASC conditions.

**Figure 3:**
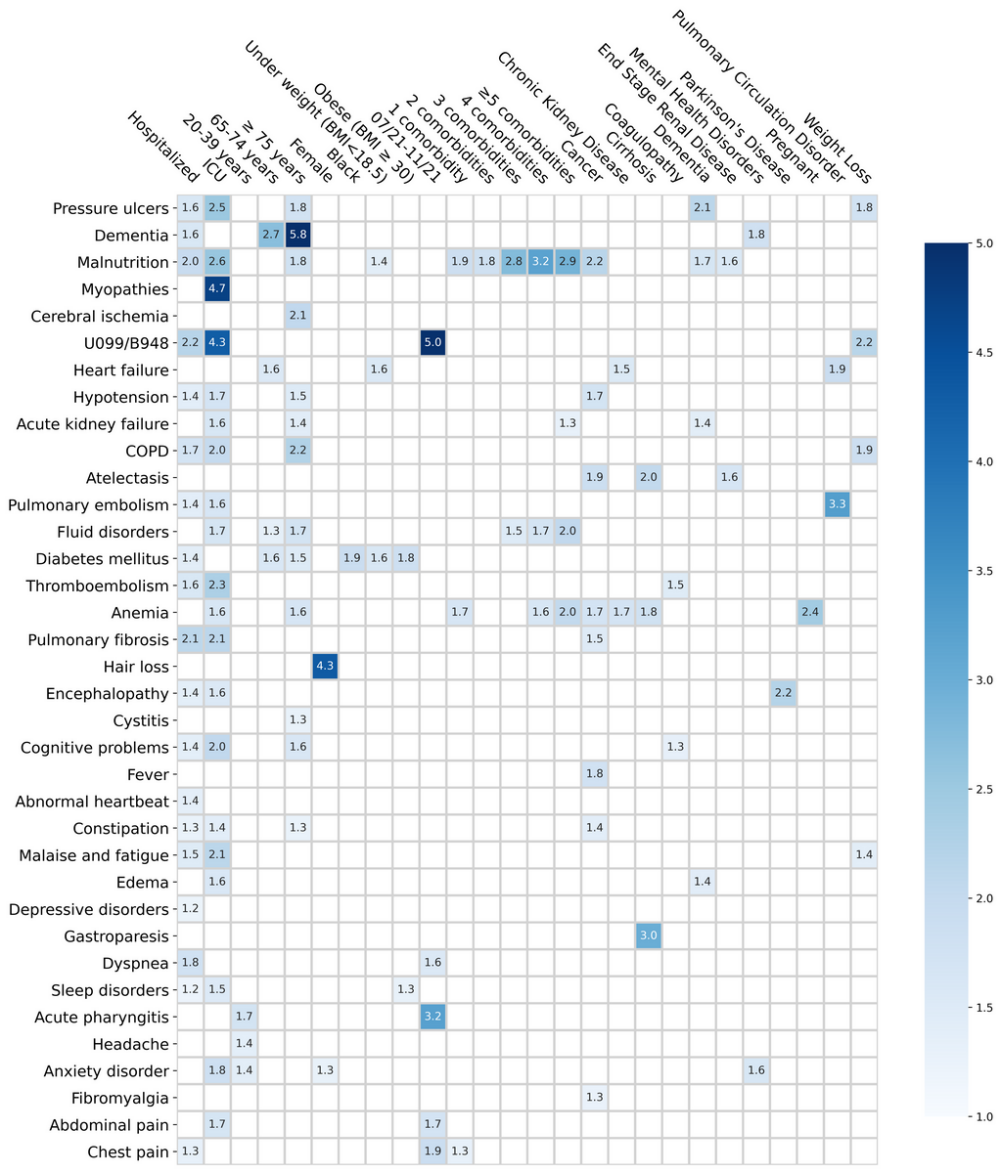
Identified risk factors associated with incident PASC conditions from the INSIGHT cohort, March 2020 to November 2021. The adjusted hazard ratios of different factors were reported. The colors represent different risk levels. ICD-10 diagnosis codes B948 (sequelae of other specified infectious and parasitic diseases) and U099 (post-COVID-19 condition, unspecified) were used to capture general post-acute sequelae diagnoses of SARS-CoV-2 infection.

Furthermore, we analyzed the associations between the covariates and the risk of developing any incident condition from our list. The unadjusted hazard ratio (HR) and fully adjusted hazard ratio (aHR) for each covariate. A covariate was identified as a potential risk factor for developing a particular condition if it satisfied the following three criteria: (1) the corresponding aHR of the covariate with respect to the target condition is larger than 1 when compared with the reference group (Method covariates section and Extended Data Table 1); (2) the association was statistically significant after multiple testing correction (p-Value < 0.000562); and (3) the associated risk was higher in SARS-CoV-2 infected patient population compared to the non-infected population. Note that criterion (3) is to guarantee the risk association we identified is not a common one that widely exists in patients without COVID-19, and the technical details on implementing this have been provided in Methods. Overall, among 35,275 enrolled SARS-CoV-2 infected patients in the INSIGHT cohort, 17,571 (49.8%) of them had at least one incident potential PASC condition (Table 1). The associations between the covariates and the risk of getting at least one PASC were summarized in Extended Data Table 1. Figure 3 depicted the associations between the identified risk factors and specific PASC conditions, which we would further elaborate on as follows.

#### The severity of acute infection.

Increased severity of the acute SARS-CoV-2 infection (according to the care settings) was associated with a higher risk of being diagnosed with new incident conditions in the post-acute period. Overall, a higher risk of getting any incident diagnosis was observed in patients who were hospitalized during the acute phase (1.29 (1.24–1.33)) or in ICU (1.40 (1.32–1.49)) compared to patients who were not hospitalized during the acute phase (as a reference group, see the Extended Data Table 1). Figure 3 further showed the associations between the acute phase severity and a range of potential PASC conditions. Specifically, compared to non-hospitalized patients, the ICU patients showed a 4.7-fold higher risk of being diagnosed with myopathy, 2.5-fold higher risk of being diagnosed with pressure ulcers, 2.3-fold higher risk of being diagnosed with thromboembolism, 2.1-fold higher risk of being diagnosed with malaise and fatigue. In addition, patients who were hospitalized or admitted to ICU during the acute phase had a higher risk of being diagnosed with general PASC codes U099/B948, with 4.3- and 2.2- fold increases compared to non-hospitalized patients.

#### Age.

Patients aged 75 or older showed an increased risk of being diagnosed with a wide range of potential PASC conditions in the post-acute infection phase, including dementia (5.8-fold higher), COPD (2.2-fold), cerebral ischemia (2.1-fold), malnutrition (1.8-fold), pressure ulcer (1.8-fold), anemia (1.6-fold), cognitive problems (1.6-fold) compared to patients were 55–64 years old (as reference). Patients with 65 to 74 years old showed an increased risk of being diagnosed with dementia (2.7-fold), heart failure (1.6-fold), and diabetes mellitus (1.6-fold) compared to reference patients. By contrast, younger patients aged 20–39 years old exhibited an increased risk of getting milder potential PASC conditions including acute pharyngitis (1.7-fold), headache (1.4-fold), and anxiety disorder (1.4-fold) than patients in the reference group.

#### Gender and Race.

Female patients exhibited a 4.3- and 1.3-fold increased risk of being diagnosed with incident hair loss and anxiety disorder in the post-acute infection period compared to male patients. Black patients exhibited a 1.9-fold increased risk of being diagnosed with incident diabetes mellitus than white patients.

#### Body Mass Index.

Patients who were underweight (BMI < 18.5 kg/m^2^) or obese (BMI ≥ 30 kg/m^2^) were at higher risk of being diagnosed with certain potential PASC conditions than those with normal BMI (BMI from 18.5 to 24.9 kg/m^2^). Specifically, underweight patients were at a 1.6-fold-increased risk of being diagnosed with heart failure, and diabetes mellitus, and a 1.4-fold-increased risk of being diagnosed with malnutrition than patients with normal BMI. Obese patients showed a 1.8-fold-increased risk of being diagnosed with diabetes mellitus and a 1.3-fold-increased risk of being diagnosed with a sleep disorder.

#### Period of infection.

We observed that patients who got infected from July 2021 to November 2021, which was dominated by the Delta variant of SARS-CoV-2^24^, showed an increased risk of being diagnosed with incident pharyngitis (3.2-fold), chest pain (1.9-fold), abdominal pain (1.7-fold), dyspnea (1.6-fold), as well as being diagnosed with general PASC symptoms and signs with the U099/B948 ICD codes (5-fold) in the post-acute infection period compared to patients got infected during March 2020 to June 2020 (the 1st wave) as the reference period.

#### Pre-existing conditions.

As shown in Fig. 3, having one or more baseline conditions was associated with a higher risk of potential PASC diagnosis including malnutrition, fluid disorders, anemia, and chest pain. Specifically, cancer patients showed increased risk in a broad list of post-acute conditions including malnutrition, atelectasis, fever, anemia, pulmonary fibrosis, constipation, and fibromyalgia compared to those without cancer diagnoses at baseline. Patients having baseline chronic kidney disease showed an increased risk of being diagnosed with heart failure and anemia. Those with baseline cirrhosis showed a 3-fold-increased risk of gastroparesis, a 2-fold-increased risk of atelectasis, and a 1.8-fold-increased risk of anemia. Those with baseline coagulopathy showed a higher risk of thromboembolism and cognitive problems. Patients with end-stage renal disease showed a higher risk of COPD and malnutrition. Those with baseline mental health disorders exhibited a higher risk of dementia and anxiety disorders in the post-acute period. Parkinson’s disease patients showed a 2.2-fold-increased risk of encephalopathy. Pregnant females showed a 2.4-fold increased risk of anemia in the post-acute period. Those with baseline pulmonary circulation disorder showed a 3.3-fold-increased risk of pulmonary embolism and a 1.9-fold-increased risk of heart failure. Patients with weight loss at baseline were at a higher risk of being diagnosed with pressure ulcers, COPD, constipation, and general PASC (with U099/B948) in the post-acute phase.

### Stratified Risk analysis

**Figure 4:**
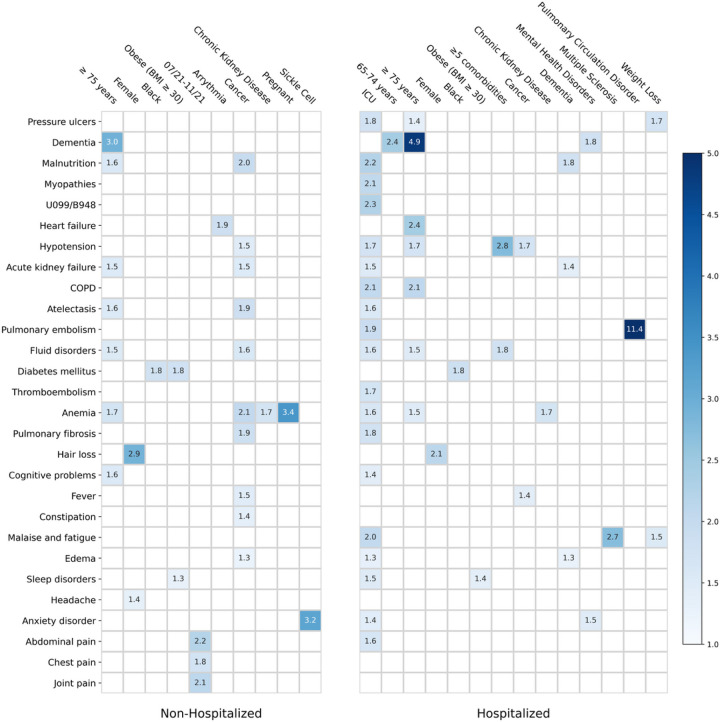
Identified risk factors associated with incident PASC conditions from the INSIGHT cohort, stratified by the hospitalization status during the acute infection, March 2020 to November 2021. The adjusted hazard ratios of different factors were reported. The colors represent different risk levels.

We further conducted stratified analyses to examine the associations between baseline factors and incident potential PASC conditions according to the care settings in the acute phase (hospitalized versus non-hospitalized). The same criteria on adjusted hazard ratio and statistical significance as we used in Fig. 3 were adopted here to identify potential risk associations, which were demonstrated in Fig. 4. Overall, certain demographic characteristics including older age, female, and black race, as well as baseline conditions including obesity and chronic kidney disease, were associated with increased risk of begin diagnosed with PASC in both non-hospitalized and hospitalized patients. There were also differences in these identified associations across the two different settings. Specifically, for patients who were not hospitalized during acute infection, we observed that baseline arrythmia was associated with a 1.9-fold increased risk of an incident diagnosis of heart failure in the post-acute period, pregnancy was associated with 3.4-fold-increased risk of incident anemia, and patients with baseline sickle cell disease showed a 3.2-fold-increased risk of being diagnosed with anxiety disorder. However, these associations were not identified among patients who were hospitalized in the acute phase. For these patients, we observed that baseline pulmonary circulation disorder was associated with an 11.4-fold-increased risk of being diagnosed with pulmonary embolism, and baseline multiple sclerosis was associated with a 2.7-fold-increased risk of being diagnosed with malaise and fatigue in the post-acute phase.

### Sensitivity analysis

We have examined the impact of the criterion on requiring the identified association to be with a higher risk in SARS-CoV-2 infected patients compared to non-infected patients. Extended Data Fig. 1 depicted the identified associations after we lifted this requirement, i.e., we only require these associations to satisfy the adjusted hazard ratio and statistical significance constraints. From the figure, we observed that more associations have been identified compared to Fig. 3, and many of these associations may not be relevant to SARS-CoV-2 infection. Taking patients with pre-existing cancer as an example, they were associated with a higher risk of being diagnosed with fluid disorders, acute kidney failure, thromboembolism, encephalopathy, edema, malaise, and fatigue in the post-acute period after SARS-CoV-2 infection. However, these associations can also be identified for non-infected cancer patients. Therefore, the excessive risk criterion is necessary for filtering out the associations that are not specific to SARS-CoV-2 infection.

We also tested to what extent the predictability of incident potential PASC conditions is affected by different machine learning models. We investigated a range of machine learning models with different complexities, including regularized logistic regression models, gradient boosting machines, and feed-forward deep neural networks. As shown in Extended Data Fig. 2, we observed little difference across the performance of these different models, and the heterogeneous predictability patterns were still observed, i.e., conditions that were difficult to predict in Fig. 2 were still with low predictive performance despite using more complex models.

Lastly, we studied if different feature engineering can impact the prediction results of different PASC conditions. Instead of using pre-defined baseline comorbidities, we leveraged a data-driven approach by using the first three digits of ICD-10 codes of all diagnoses and all medications in RxNorm codes at the ingredient level in the baseline period to predict PASC. We reported the predictive performance of different machine learning models using this large set of features in Extended Data Fig. 3, which does not show big differences compared to the performance in Extended Data Fig. 2 or Fig. 2, and the heterogeneous predictability patterns remain the same.

### Validation by the OneFlorida + Cohort

To assess the generalizability of our findings, we replicated our analyses on the OneFlorida + cohort as an independent validation. The OneFlorida + cohort included 22,341 adult patients with lab-confirmed SARS-CoV-2 infection and 177,010 non-infected as control patients (See inclusion cascade in Fig. 1). We summarized the prediction performance of different potential PASC conditions with regularized Cox model in Extended Data Fig. 4 and the identified risk associations in Extended Data Fig. 5. From Extended Data Fig. 4 we again observed the heterogeneous predictability of different conditions as has been observed in Fig. 2, and the more predictable conditions (with c-index > 0.8, such as malnutrition, COPD, dementia, and acute kidney failure) and less predictable (with c-index around or below 0.6, such as fatigue, anxiety, sleep disorders, and depression) remained the same. Similarly, the risk associations shown in Extended Data Fig. 4 are consistent with the risk associations shown in Fig. 3. Hospitalization and ICU admission in the acute infection phase were associated with a diverse set of incident diagnoses in the post-acute infection phase. We still observed the risk associations between older age and dementia (5.4-fold increased risk), female and hair loss (2.2-fold increased risk), black race, and diabetes (1.5-fold increased risk). Infection confirmation from July to November 2021 was associated with a 1.7-fold increased risk of being diagnosed with general PASC symptoms and signs (the U099/B948 ICD code).

## Discussion

In this paper, we conducted a systematic study on the predictability of a wide range of potential PASC conditions as well as their associated risk factors using the EHR data from two large-scale PCORnet clinical research networks, INSIGHT, and OneFlorida+. Compared with existing research on this topic which was mostly based on patient-reported symptoms^12,25^, our study was based on routinely collected EHR data from large patient populations.

We investigated the predictability of different potential PASC diagnoses using patient demographics, prior conditions, and care settings in the acute phase. Different types of machine learning models, including linear models, tree-based models, and deep learning models were tested. Following prior research on PASC^3,26^, we focused on newly incident conditions in the post-acute infection period in this study. We have built a comprehensive list of diagnoses based on a prior study by Al-Aly et al.^3^, with further refinements from our clinician team. Different from existing relevant studies that treated PASC as a holistic concept^3,26^, we have explored the predictability and potential risk factors of each individual condition, as there had been abundant evidence suggesting PASC was a highly heterogeneous condition affecting multiple organ systems^3,14^.

The results from regularized Cox regression were summarized in Fig. 2, which suggested that different conditions were associated with different predictabilities in the INSIGHT cohort. Conditions such as stroke, heart failure, and kidney failure were more predictable. These conditions are with clear diagnostic criteria according to the underlying disease etiologies and are more likely to be severe COVID complications. Pressure ulcers was also highly predictable, but it was more likely due to prolonged hospital stay during the acute phase of SARS-CoV-2 infection^27^. General PASC symptoms and signs with the U099/B948 codes were also associated with good prediction performance, which is consistent with prior studies^28^. One potential reason was that these codes were relatively new, and the clinicians might be cautiously using them only when the symptoms and signs were typical. Conditions such as headache, dizziness, chest pain, joint pain, anxiety, and depressive disorders, were more difficult to predict. These conditions are most subjective to diagnose, more similar to patient-reported symptoms, and cannot be explained by alternative disease etiologies. The prediction performance obtained from more complex machine learning models did not make such differences, as evidenced by Extended Data Fig. 2. In addition, we have replicated the predictive modeling analysis on the OneFlorida + cohort, and the results summarized in Extended Data Fig. 4 were highly consistent with the conclusions obtained from the INSIGHT cohort.

With fully adjusted analysis, we examined the statistical associations between a broad list of covariates including demographics, pre-existing conditions, and severities in the acute phase of SARS-CoV-2 infection according to care settings and each potential PASC condition. For a covariate to be considered as a potential risk factor of a specific condition, we required its corresponding adjust hazard ratio (aHR) to be larger than 1 and statistically significant. We further required the estimated aHR value to be larger in patients who were infected by SARS-CoV-2 compared to the non-infected patients, in this way associations that may not be attributed to COVID-19 can be filtered. Figure 3 and Extended Data Fig. 5 summarized the identified risk associations from the INSIGHT and OneFlorida + cohorts. Both figures showed that hospitalizations and admissions to ICU during the acute infection phase were associated with a broad set of incident conditions in the post-acute infection phase, including pressure ulcers, heart failure, acute kidney failure, COPD, etc., which suggested that these conditions could be related to either severe acute COVID complications or acute care processes. Older age (> = 75 years) was also found to be a potential risk factor for many of these conditions. Black patients were at higher risk of being diagnosed with incident diabetes. These discoveries were consistent with the conclusions from prior studies^29,30^. Other notable risk associations consistently identified from both cohorts include higher baseline comorbidity burden and fluid disorder, baseline obesity and sleep disorder, as well as baseline end-stage renal disease and malnutrition. Some associations should be interpreted more cautiously. For example, baseline pulmonary circulation disorder was consistently identified as a risk pulmonary embolism, but the two conditions are highly correlated with each other, and this association could just be due to the ICD coding and grouping. Another example was baseline pregnancy and anemia, as anemia is the most common hematologic problem in pregnancy^31^. However, there were also studies suggesting that SARS-CoV-2 infection during pregnancy can further exacerbate iron deficiency anemia due to hyperinflammation during the acute infection phase^32^.

There were several strengths of our study. First, we studied a comprehensive set of associations between 89 factors and 44 incident PASC conditions in two large EHR cohorts. To our knowledge, this is one of the largest studies on predictive modeling and risk factor analysis for PASC. Second, we derived our primary results from INSIGHT and did a validation study on OneFlorida+, which validated the generalizability of our findings. Third, we have tested the prediction performance of different machine learning models on both a narrow and broad list of covariates, which further validates the robustness of our conclusions. Finally, we ruled out potential background associations by requiring the adjusted hazard ratio value of the identified association estimated from the patients who were infected by SARS-CoV-2 to be larger than the value estimated from patients who are not infected by SARS-CoV-2.

Our study had several limitations. Our analysis was based on EHR data, which would miss the information from patients who did not visit the hospitals within the CRNs. We only considered newly incident individual conditions in the post-acute period but did not explore conditions that were prolonged, worsened, or relapsed before and after COVID-19 infection, as well as condition clusters or subphenotypes. Vaccine information was not incorporated in our study due to its incompleteness in the EHR records, and we are working on adding other data sources (e.g., state registry data) to make the information more robust. In addition, our analyses did not cover the recent Omicron wave due to the availability of the data.

In conclusion, we used two large-scale clinical research networks, INSIGHT and OneFlorida + to identify risk factors associated with newly incident PASC conditions and to develop predictive models to identify those who are at risk of these conditions. Our results highlight that several predictive PASC diagnoses are associated with severity in the acute phase. However, less predictable PASC diagnoses represent an ongoing challenge that may not respond to other measures that decrease the severity of acute COVID.

## Figures and Tables

**Table 1:** Description of the INSIGHT lab-confirmed SARS-Cov-2 positive cohort with the number of at least one incident PASC diagnosis by patient characteristics, INSIGHT, March 2020 to November 2021a.

Characteristics	Number of Patients with Confirmed SARS-CoV-2 Infection (columns %)	Number of Patients with Confirmed SARS-CoV-2 Infection and at least One Incident PASC Condition (% within stratum)
**Total**	35,275	17,571 (49.8)
**The Severity of Acute Infection — no. (%)**		
Not hospitalized	22,148 (62.8)	9,809 (44.3)
Hospitalized w/o ICU	11,480 (32.5)	6,611 (57.6)
ICU	1,647 (4.7)	1,151 (69.9)
**Age group — no. (%)**		
20–<40 years	9,529 (27.0)	3,875 (40.7)
40–<55 years	7,975 (22.6)	3,850 (48.3)
55–<65 years	6,965 (19.7)	3,606 (51.8)
65–<75 years	5,712 (16.2)	3,170 (55.5)
75 + years	5,094 (14.4)	3,070 (60.3)
**Sex — no. (%)**		
Female	20,686 (58.6)	10,295 (49.8)
Male	14,586 (41.3)	7,275 (49.9)
**Race — no. (%)**		
Asian	1,736 (4.9)	799 (46.0)
Black	7,791 (22.1)	4,029 (51.7)
White	12,233 (34.7)	5,896 (48.2)
Other	9,844 (27.9)	5,208 (52.9)
Missing	3,671 (10.4)	1,639 (44.6)
**Ethnic group - no. (%)**		
Hispanic	10,658 (30.2)	5,789 (54.3)
Not Hispanic	20,838 (59.1)	10,305 (49.5)
Other/Missing	3,779 (10.7)	1,477 (39.1)
**Median area deprivation index (IQR) — rank**		
ADI1–19	25,611 (72.6)	12,715 (49.6)
ADI20–39	7,891 (22.4)	3,962 (50.2)
ADI40–59	1,126 (3.2)	550 (48.8)
ADI60–79	162 (0.5)	80 (49.4)
ADI80–100	485 (1.4)	264 (54.4)
**No. of hospital visits in the past 3 year — no. (%)**		
Inpatient visits 0	25,717 (72.9)	12,368 (48.1)
Inpatient visits 1–2	6,805 (19.3)	3,677 (54.0)
Inpatient visits > = 3	2,753 (7.8)	1,526 (55.4)
Outpatient visits 0	1,570 (4.5)	1,077 (68.6)
Outpatient visits 1–2	3,327 (9.4)	1,677 (50.4)
Outpatient visits > = 3	30,378 (86.1)	14,817 (48.8)
Emergency visits 0	20,351 (57.7)	9,589 (47.1)
Emergency visits 1–2	9,518 (27.0)	5,053 (53.1)
Emergency visits > = 3	5,406 (15.3)	2,929 (54.2)
**Body Mass Index**		
BMI: <18.5 underweight	6,419 (18.2)	3,685 (57.4)
BMI: 18.5–<25 normal weight	6,431 (18.2)	3,272 (50.9)
BMI: 25–<30 overweight	8,116 (23.0)	4,001 (49.3)
BMI: >=30 obese	9,751 (27.6)	4,918 (50.4)
BMI: missing	4,558 (12.9)	1,695 (37.2)
**Smoking**		
Smoker: never	1,827 (5.2)	925 (50.6)
Smoker: current	842 (2.4)	404 (48.0)
Smoker: former	781 (2.2)	404 (51.7)
Smoker: missing	31,825 (90.2)	15,838 (49.8)
**Index periods of patients — no. (%)**		
03/20 – 06/20	11,235 (31.8)	5,971 (53.1)
07/20 – 10/20	2,018 (5.7)	1,001 (49.6)
11/20 – 02/21	14,637 (41.5)	7,256 (49.6)
03/21 – 06/21	5,573 (15.8)	2,802 (50.3)
07/21 – 11/21	1,812 (5.1)	541 (29.9)
**Pre-existing conditions — no. (%)** ^b^		
No comorbidity	11,072 (31.4)	4,714 (42.6)
1 comorbidity	6,330 (17.9)	3,037 (48.0)
2 comorbidities	3,741 (10.6)	1,877 (50.2)
3 comorbidities	3,069 (8.7)	1,601 (52.2)
4 comorbidities	2,410 (6.8)	1,334 (55.4)
>=5 comorbidities	8,653 (24.5)	5,008 (57.9)
Alcohol Abuse	1,018 (2.9)	566 (55.6)
Anemia	4,862 (13.8)	2,717 (55.9)
Arrhythmia	5,350 (15.2)	3,072 (57.4)
Asthma	3,950 (11.2)	2,179 (55.2)
Autism	47 (0.1)	22 (46.8)
Cancer	3,616 (10.3)	2,082 (57.6)
Chronic Kidney Disease	5,126 (14.5)	2,995 (58.4)
Chronic Pulmonary Disorders	6,209 (17.6)	3,511 (56.5)
Cirrhosis	582 (1.6)	374 (64.3)
Coagulopathy	2,511 (7.1)	1,502 (59.8)
Congestive Heart Failure	3,682 (10.4)	2,203 (59.8)
COPD	1,726 (4.9)	1,027 (59.5)
Coronary Artery Disease	4,658 (13.2)	2,652 (56.9)
Cystic Fibrosis	13 (0.0)	8 (61.5)
Dementia	1,404 (4.0)	884 (63.0)
Diabetes Type 1	435 (1.2)	247 (56.8)
Diabetes Type 2	7,681 (21.8)	4,310 (56.1)
Down’s Syndrome	30 (0.1)	14 (46.7)
End Stage Renal Disease on Dialysis	1,573 (4.5)	913 (58.0)
Epstein-Barr and Infectious Mononucleosis (Mono)	33 (0.1)	15 (45.5)
Hemiplegia	440 (1.2)	253 (57.5)
Herpes Zoster	255 (0.7)	133 (52.2)
HIV	586 (1.7)	308 (52.6)
Hypertension	13,796 (39.1)	7,687 (55.7)
Hypertension and Type 1 or 2 Diabetes Diagnosis	6,426 (18.2)	3,649 (56.8)
Inflammatory Bowel Disorder	367 (1.0)	182 (49.6)
Lupus or Systemic Lupus Erythematosus	290 (0.8)	157 (54.1)
Mental Health Disorders	3,682 (10.4)	2,129 (57.8)
Multiple Sclerosis	187 (0.5)	97 (51.9)
Obstructive sleep apnea	2,105 (6.0)	1,138 (54.1)
Other Substance Abuse	2,225 (6.3)	1,238 (55.6)
Parkinson’s Disease	222 (0.6)	124 (55.9)
Peripheral vascular disorders	2,562 (7.3)	1,459 (56.9)
Pregnant	1,608 (4.6)	561 (34.9)
Pulmonary Circulation Disorder	1,345 (3.8)	831 (61.8)
Rheumatoid Arthritis	544 (1.5)	308 (56.6)
Seizure/Epilepsy	790 (2.2)	455 (57.6)
Severe Obesity (BMI > = 40 kg/m^2^)	2,519 (7.1)	1,338 (53.1)
Sickle Cell	255 (0.7)	134 (52.5)
Weight Loss	1,603 (4.5)	964 (60.1)
Prescription of Corticosteroids	4,999 (14.2)	2,695 (53.9)
Prescription of Immunosuppressant drug	2,110 (6.0)	1,086 (51.5)

a. The SARS-CoV-2 positive patients were identified by polymerase chain reaction (PCR) test or antigen test. The percentage may not sum up to 100 because of rounding.

b. Coexisting conditions existed if two records in the 3 years prior to the index event. See detailed phenotyping codes in the appendix. SLE: Systemic Lupus Erythematosus; COPD: Chronic obstructive pulmonary disease.

## Data Availability

The INSIGHT data can be requested through https://insightcrn.org/. The OneFlorida+ data can be requested through https://onefloridaconsortium.org. Both the INSIGHT and the OneFlorida+ data are HIPAA-limited. Therefore, data use agreements must be established with the INSIGHT and OneFlorida+ networks.
